# Surface roughness of titanium disks influences the adhesion, proliferation and differentiation of osteogenic properties derived from human

**DOI:** 10.1186/s40729-020-00243-5

**Published:** 2020-08-25

**Authors:** Maria Alejandra Frias Martinez, Ísis de Fátima Balderrama, Paula Stephania Brandão Hage Karam, Rodrigo Cardoso de Oliveira, Flávia Amadeu de Oliveira, Carlos Roberto Grandini, Fábio Bossoi Vicente, Andreas Stavropoulos, Mariana Schutzer Ragghianti Zangrando, Adriana Campos Passanezi Sant’Ana

**Affiliations:** 1 Department of Prosthodontics and Periodontics, Discipline of Periodontics, School of Dentistry at Bauru, University of São Paulo, Bauru, SP 17012-901 Brazil; 2 Department of Periodontology, Faculty of Odontology, Malmö University, Malmö, Sweden; 3 Department of Biological Sciences, School of Dentistry at Bauru, University of São Paulo, Bauru, SP Brazil; 4 Anelasticity and Biomaterials Laboratory, São Paulo State University, Bauru, SP Brazil

**Keywords:** Fibroblasts, Titanium, Surface analysis, Cell adhesion, cell proliferation

## Abstract

**Purpose:**

The aim of this study was to investigate the response of osteogenic cell lineage and gingival fibroblastic cells to different surface treatments of grade IV commercially pure Titanium (cpTi) disks.

**Material and methods:**

Grade IV cpTi disks with different surfaces were produced: machined (M), sandblasting (B), sandblasting and acid subtraction (NP), and hydrophilic treatment (ACQ). Surface microtopography characteristics and chemical composition were investigated by scanning electron microscopy (SEM) and energy dispersive x-ray spectrometry (EDS). Adhesion and proliferation of SC-EHAD (human surgically-created early healing alveolar defects) and HGF-1 (human gingival fibroblasts) on Ti disks were investigated at 24 and 48 h, and osteogenic differentiation and mineralization were evaluated by assessing alkaline phosphatase (ALP) activity and alizarin red staining, respectively.

**Results:**

No significant differences were found among the various surface treatments for all surface roughness parameters, except for skewness of the assessed profile (Rsk) favoring M (*p* = 0.035 ANOVA). M disks showed a slightly higher (*p* > 0.05; Kruskal-Wallis/Dunn) adhesion of HGF-1 (89.43 ± 9.13%) than SC-EHAD cells (57.11 ± 17.72%). ACQ showed a significantly higher percentage of SC-EHAD (100%) than HGF-1 (69.67 ± 13.97%) cells adhered at 24 h. SC-EHAD cells expressed increased ALP activity in osteogenic medium at M (213%) and NP (235.04%) surfaces, but higher mineralization activity on ACQ (54.94 ± 4.80%) at 14 days.

**Conclusion:**

These findings suggest that surface treatment influences the chemical composition and the adhesion and differentiation of osteogenic cells in vitro.

**Clinical relevance:**

Hydrophilic surface treatment of grade IV cpTi disks influences osteogenic cell adhesion and differentiation, which might enhance osseointegration.

## Introduction

Osseointegration was originally defined as a direct union between vital bone and a functioning metal implant at optical microscopy [1]. Since then, dental implants have significantly evolved, especially in surface treatment, aiming at improving the quality and/or speed of osseointegration [2, 3]. Osseointegration is influenced by varying parameters, including material, design, surface properties, surgical technique, and bone quality [1, 4]. A modification of implant surface topography has been considered as an essential parameter contributing to the success of dental implants [2]. A major part of implantology research focuses on the development of surface modifications that would be able of improving the biologic characteristics of titanium [5, 6]. Considering that, new surfaces of dental implants were developed to improve biological cell responses, guiding the differentiation of stem cells in osteoblasts and enhancing osseointegration [7]. Material biocompatibility is intimately related to cell behavior [8, 9]. Implant surface microtopography influences adhesion, proliferation, differentiation, and extracellular matrix synthesis by osteoblasts and other cells [9–15]. Surface roughness also influences the behavior of osteoprecursor cells by stimulating proliferation and inducing differentiation into osteoblasts [16–19] and bone growth at implant threads, which may affect the process of osseointegration [6, 20–26]. Popular treatments of implant surfaces, increasing roughness, include blasting and/or acid etching, as well as addition of nanoparticles and rising under protection with N2, followed by storage in NaCl solution [7, 27]. These modifications affect cell behavior, improving the adsorption of proteins, and favoring osteoblastic cells differentiation [7, 28, 29]. The effects of titanium surface topography in the behavior of osteoblasts are associated to adhesion-related cell function [7, 30]. Recently, the granulation tissue present in surgically created bone defects in the jaws of humans after 21 days of healing was isolated and characterized in vitro for the first time, and this granulation tissue removed from human surgically created early healing alveolar defects (SC-EHAD cells) demonstrated osteogenic properties [31]. Cells exhibited a spindle-shaped morphology at earlier passages, changing to a cuboidal one at later passages. Alkaline phosphatase (ALP) activity and mineralization were observed both in conventional and osteogenic medium. Fresh samples of SC-EHAD tissue exhibited CD34− and CD45−phenotypes, while SC-EHAD cells at later passages exhibited, besides that, a CD105−, CD166−, and collagen type I+ phenotype. These findings suggest that SC-EHAD is a possible source of progenitor cells [31]. However, the properties of this human cell lineage when cultivated on different implant surfaces are yet unknown. Considering that, the aim of this study is to investigate the response of SC-EHAD cells to different grade IV cpTi disks surface treatments.

## Material and methods

### Titanium discs and surface preparation

Grade IV cp Ti disks, 6.0 mm × 2.0 mm (Neodent®, Curitiba, Brazil) with the following surface treatments, were used:

1. Machined (M)
2. Sandblasted (B—subtraction with silicon, aluminum, and titanium oxide creating abrasion on disks surface)
3. Sandblasted and acid etched (combination: hydrofluoric, nitric, and sulfuric acid) (NP—Neoporos®, surface)
4. Sandblasted, acid etched, and immersed in 0.9% sodium chloride (ACQ—Acqua®, surface), were used.

### Surface analysis

Disks surfaces were examined by scanning electron microscopy, at × 500 magnification in a high resolution scanning electron microscopy (Machining Technology Laboratory, School of Engineering, São Paulo State University. Bauru, Brazil). Roughness characteristics were examined in SEM photomicrographs by SurfCharJ plugin (available for download at: http://imagej.nih.gov/ij/), which measures roughness parameters according to ISO 4287/2000: Ra (arithmetical mean deviation), Rq (root mean square deviation), Rku (kurtosis of the assessed profile), Rsk (skewness of the assessed profile), Rv (lowest valley), Rp (highest peak), and Rt (total height of the profile). The chemical composition of titanium disks was investigated by energy dispersive x-ray detector (EDS). One sample of each group was analyzed in three different locations to detect the chemical composition in order to detect possible differences between regions.

### Cell culture

After approval of the Committee of Ethics in Research (CAAE 32274414900005417), SC-EHAD cells were obtained from two systemically healthy individuals, non-smoking, 40 and 45 years old males who signed the consent form for newly forming bone graft technique during periodontal treatment of deep infrabony periodontal pockets and furcation lesions, as described the treatment protocol by Passanezi and co-authors in 1989 [32]. Briefly, a 10-mm height × 3.5 mm diameter surgical defect was created at the alveolar ridge with a cylindrical diamond bur under copious irrigation, as previously method described [31–33]. After 21 days [32], defects were re-opened, the granulation tissue present in the healing defects was collected with a Lucas curette, and a portion of the material was transferred to a Falcon tube containing Dulbecco’s minimal essential medium (DMEM, Sigma-Aldrich, USA), 20% fetal bovine serum (FBS, Sigma-Aldrich, USA), 200 U/mL G potassium penicillin, 200 mg/mL streptomycin sulfate, and 20 μg/mL amphotericin B (Sigma-Aldrich, USA), allowing the establishment of primary culture of osteoblasts after centrifugation of the fine dissected fragments for 3 min and positioning in 25 cm^2^ tissue flasks containing DMEM supplemented with 20% FBS and 2% antibiotic-antimycotic solution. The option to collection of SC-EHAD after 21 days after creation of defect was based on the clinical applications already proposed [32].

Human gingival fibroblasts (HGF-1), used as positive controls for comparisons, were obtained from two systemically healthy, non-smoking volunteers submitted to a gingivectomy by internal bevel incision (*n* = 1) and a free gingival graft (*n* = 1) procedure. The tissue removed during surgical procedures was positioned in Falcon tubes containing DMEM (Eagles minimal essential medium) supplemented with 20% FBS (fetal bovine serum) and 2% antibiotic-antimycotic solution, and transported to cell culture lab to the establishment of primary culture. Tissue fragments were finely dissected in Petri dishes containing saline buffer (PBS) and 2% antibiotic-antimycotic solution. Cell separation was performed by mechanical-enzymatic process by submersion of tissue fragments in trypsin solution at 37 ^°^C followed by centrifugation for 3 min. This process was repeated once, and the resultant cell pellet was re-suspended in DMEM supplemented with 20% FBS and 2% antibiotic-antimycotic solution. Cells were allowed to expand in humidified atmosphere containing 5% CO_2_ at 37 ^°^C until reaching subconfluency (approximately 80% of cultivable area covered by cells); when cells were detached with trypsin solution (Sigma-Aldrich, USA) and transferred to progressively greater tissue flasks until experimental procedures were performed, the cell sources were pooled to HGF-1 lineage group.

### Adhesion and proliferation assays

5 × 10^4^ cells in 50 μl of DMEM were platted on 5 disks/group (SC-EHAD or HGF-1) in 96-well plates. Four hours after platting, 200 μl of culture medium was added in each well, completely covering the disks. Samples were fixated after 24 h (adhesion assay) and 48 h (proliferation assay) with Karnovsky solution (6% glutaraldehyde and 4% paraphormaldehyde in 0.2 M cadodylate buffer) and post-fixated with 2% osmium tetroxide in cacodylate buffer at 4 ^°^C for 2 h. After dehydration in graded alcohols, samples were immersed in 100% hexametildisilazane (HMDS) at room temperature for 24 h, air-dried, and sputter-coated with gold for examination by scanning electron microscopy (SEM). From each specimen, 2 photomicrographs were obtained (central and a randomly selected peripheric area) at × 500 magnification.

Each photomicrograph was coded and analyzed by a blinded examiner for assessing the area covered by cells. After calibration of the image size, a grid was superposed on SEM images in an image analysis software (ImageJ®, NIH, Bethesda, USA) software, and the area without cells was determined and expressed as % of the total disk area. Additionally, cell adhesion and proliferation were analyzed on each image, by a blinded examiner, using an index from 0–5 (Table 1).

**Table 1:** Index of cell adhesion and proliferation

Score	Characteristics
0	No cells adhered
1	< 50% area covered by cells
2	50–<75% area covered by cells
3	75–100% area covered by cells
4	100% area covered by cells + formation of a second cell layer

### Differentiation and mineralization assays

SC-EHAD and HGF-1 cells were cultured on the disks and plastic (negative control) at an initial density of 7 × 10^3^ cells in 24-well plates. After initial adhesion (4 h) in standard medium, cells were cultivated in standard and osteogenic medium. The osteogenic medium was obtained by supplementation of standard medium with 10 mM β-glicerophosphate (Sigma, USA) and 50 μg/ml ascorbic acid. The assays were performed in triplicates. Alkaline phosphatase (ALP) was determined in pool after 14 days. The culture medium was discarded, and cells were twice washed with saline solution and lysed by adding 100× Triton solution. ALP was observed at lysed cells using 25 μl of the sample added in 200 μl of p-nitrophenol phosphate. Total protein measurements were performed according to Bradford method. The optical density was read at 405 nm in a spectrophotometer (FLUOstar Optima microplate reader, BMG, Leicester, UK). ALP activity (U/ml) was normalized according to protein measurements for each sample.

Alizarin red staining was performed after 14 and 28 days of culture of SC-EHAD and HGF-1 cells on the different substrates in triplicates at initial density of 5 × 10^4^ cells in osteogenic and standard culture media, as described before. The culture medium was discarded; confluent cell layers were washed with PBS at 37 ^°^C, fixated with 4% formalin solution, and again washed with PBS. The disks were stained with alizarin red S (2%, pH 4.2, Merck). Images of the disks were captured with stereomicroscope and processed in Image J to determine the percentage of area showing mineralized nodules stained by alizarin red.

### Statistical analysis

Data were analyzed in Graph Pad Prism 6.0 for Mac, adopting a significance level of 5% (*α* = 0.05) for all tests. Comparisons of roughness characteristics among groups were performed by ANOVA post hoc Tukey. The percentage of area covered by cells and index of cell adhesion and proliferation were compared by non-parametric Kruskal-Wallis post hoc Dunn at the different periods. Inter-group analysis of alizarin red staining was performed by non-parametric Kruskal-Wallis post hoc Dunn, and intra-group analysis was performed by non-parametric Wilcoxon test. Differences in ALP activity and mineralization assay were assessed with the Wilcoxon test.

## Results

### Surface characteristics

**Fig. 1:**
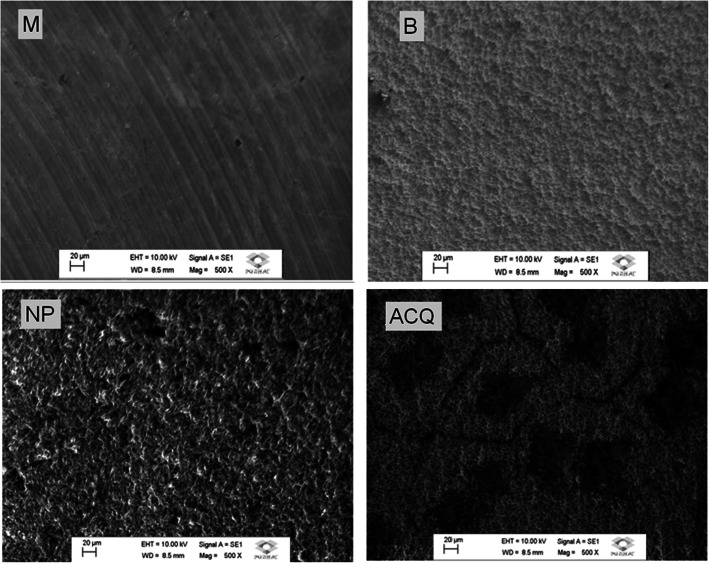
SEM photomicrographs. Titanium disks with machined (M), sandblasted (B), Neoporos® (NP), and Acqua® (ACQ) surfaces

**Fig. 2:**
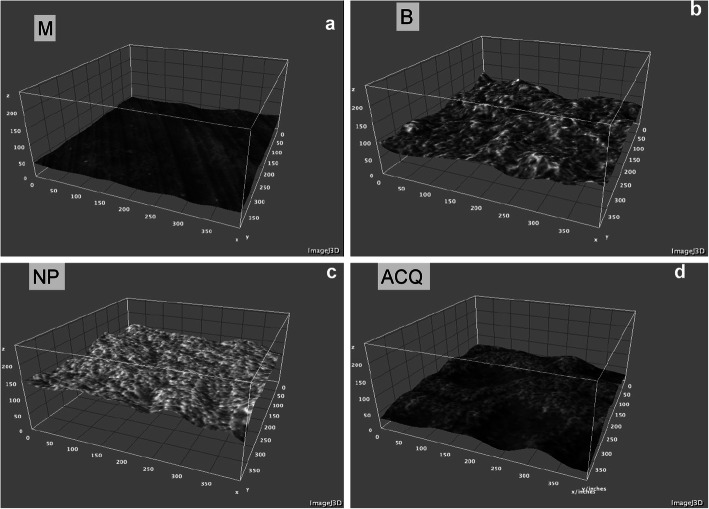
3D surface plot. **a** M, **b** B, **c** NP, and **d** ACQ groups obtained by Interactive 3D, Surface Plot plugin (ImageJ, NIH, USA)

**Fig. 3:**
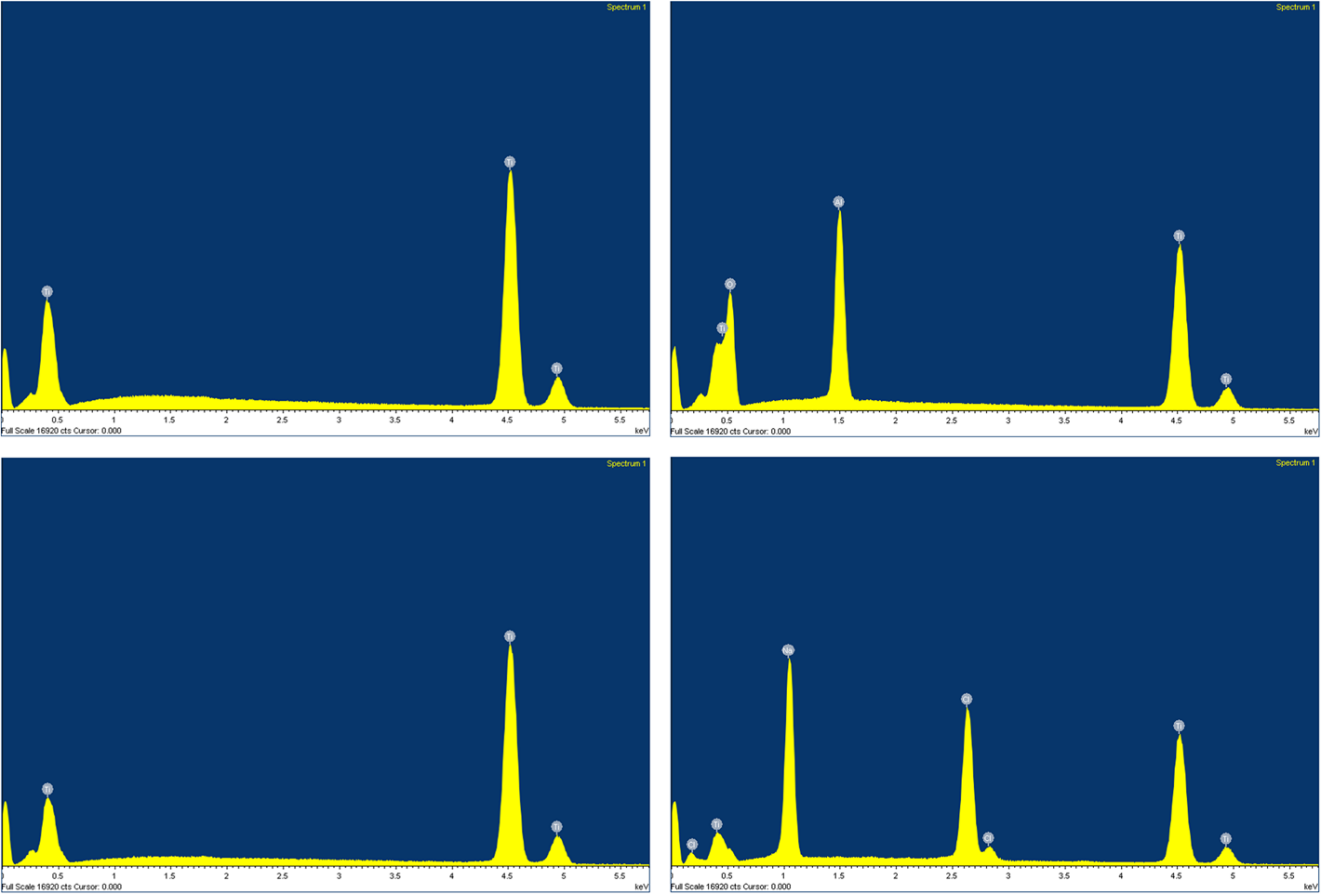
EDS analysis. Chemical composition of titanium disks with different surface treatment. **a** M. **b** B. **c** NP. **d** ACQ

No differences in roughness were found among groups regarding all parameters, except for Rsk (*p* < 0.05; ANOVA post hoc Tukey; Table 2). Surface characteristics are presented in SEM photomicrographs (Fig. 1), while the 3D-plot surface characteristics of the different surfaces are illustrated in Fig. 2. The chemical composition performed by EDS was resulted in weight percentage (weight %), and Fig. 3 showed each surface found. M and NP surface were composed by titanium only (100%); B surface showed the presence of titanium (62.13%), aluminum (10.78%), and oxygen (27.09%); and ACQ surface showed presence of titanium (61.99%), sodium (15.84%), and Chlorum (22.17%).

**Table 2:** Roughness parameters analyzed by SurfCharJ plugin (ImageJ, NIH, USA) [Sample length 100 μm, surface leveling]

	M	B	NP	ACQ
**Rq**	0.1289	0.1482	0.1956	0.1616
**Ra**	0.0983	0.1104	0.1574	0.1252
**Rsk**	1.9698*	1.5975	0.6909	1.3361
**Rkv**	13.4801	3.5427	0.1222	2.4683
**Rv**	– 0.2893	– 0.2174	– 0.4170	– 0.2591
**Rp**	1.9496	0.8084	0.6484	1.1277
**Rt**	2.2388	1.0258	1.0653	1.3867
**Rc**	– 0.0046	0.0012	0.0009	– 0.0030

*Significant differences between M, B, NP, and ACQ (*p* = 0.035)

### Adhesion and proliferation assays

#### 24 hours

**Fig. 4:**
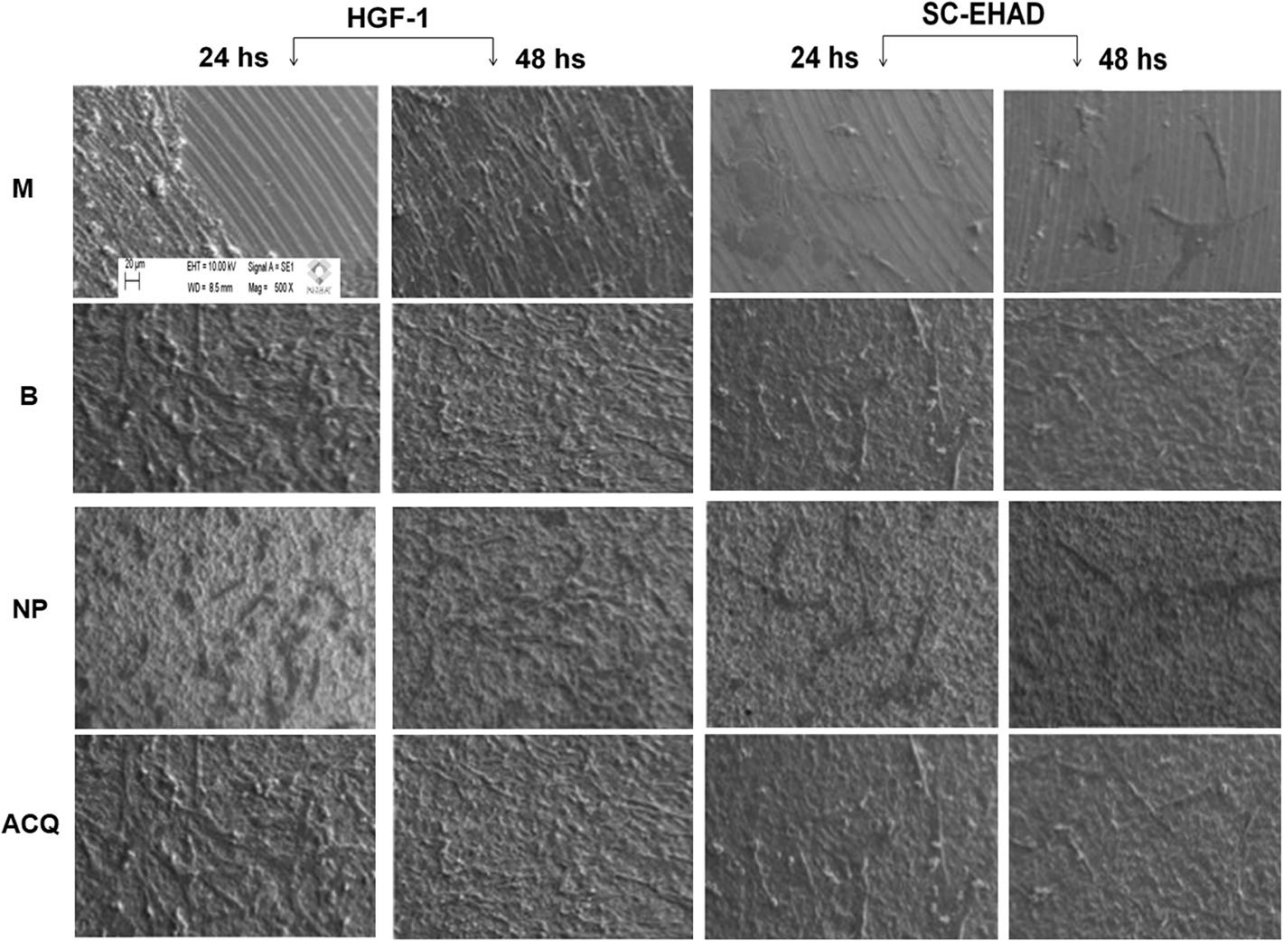
SEM photomicrographs of HGF and SC-EHAD cell adhesion and proliferation. SEM images of HGF-1 and BG-1 cells cultivated on M, B, N,P and ACQ surfaces after 24 and 48 h of culture (× 500 magnification)

**Fig. 5:**
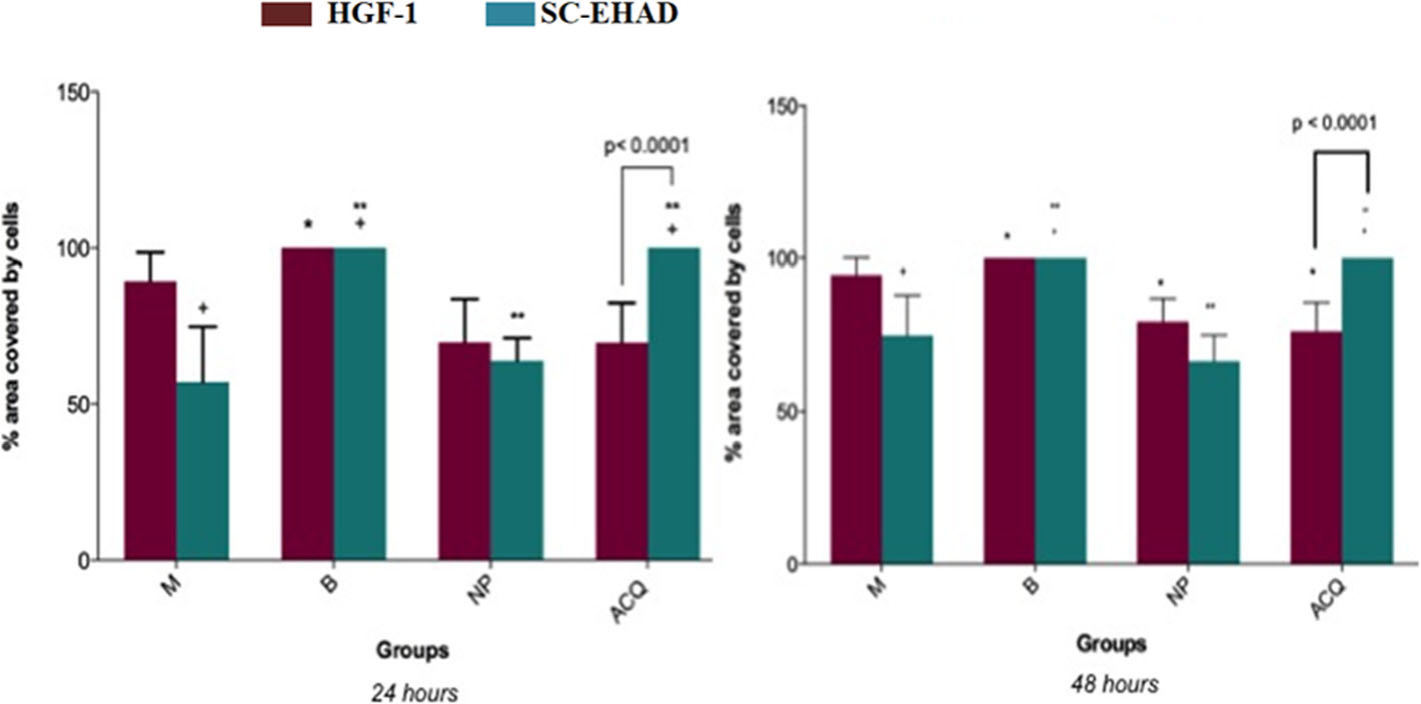
Percentage area of the disks covered by cells (mean ± standard deviation) at 24 and 48 h, according to groups. Equal symbols in columns HGF-1 represent *significant differences between groups B and NP, ACQ, and M (*p* < 0.01; Kruskal-Wallis post hoc Dunn). Equal symbols in columns SC-EHAD represent ^+^significant differences between M and groups B and ACQ (*p* < 0.001; Kruskal-Wallis post hoc Dunn); ^**^significant differences between NP and groups B and ACQ (*p* < 0.001; Kruskal-Wallis post hoc Dunn)

ACQ and B surfaces were covered at 100% by SC-EHAD cells (Figs. 4 and 5). M surfaces showed 57.11% ± 17.72%, and NP surfaces showed 63.87% ± 7.23% area covered by SC-EHAD. ACQ surfaces showed 69.67% ± 13.97% area covered by HGF-1, and B surfaces were covered at 100%. M surfaces showed 89.43% ± 9.13% and NP surfaces showed 69.77% ± 13.97% area covered by HGF-1.

#### 48 hours

Significant differences in the area covered by cells were found only for SC-EHAD on ACQ surfaces (*p* = 0.03; Wilcoxon) when compared with areas covered by HGF-1 (Fig. 5). Significant differences between the area occupied by cells at 24 and 48 h were observed only for SC-EHAD on ACQ surfaces (*p* = 0.03; Wilcoxon). A significant smaller area occupied by HGF-1 (*p* < 0.01; Kruskal-Wallis) and SC-EHAD (*p* < 0.005; Kruskal-Wallis) was observed in B than in NP and ACQ (Fig. 5).

### Adhesion and proliferation index

#### SC-EHAD

A higher prevalence of score 4 was observed for groups B and ACQ compared to groups M (median 2.0) and NP (median 2.0) at 24 h. At 48 h, significant differences were found between M (median 2.5) and NP (median 2.0) compared to B (median 4.0) and ACQ (median 4.0) surfaces. Significant differences between scores of HGF-1 and SC-EHAD cells cultivated on ACQ surfaces were found at 24 and 48 h. No significant intra-group differences were found between 24 and 48 h (Table 3).

**Table 3:** Adhesion (24 h) and proliferation (48 h) index of HGF-1 and SC-EHAD cells at the different surfaces [median (mean ± standard-deviation)]

	HGF-1		SC-EHAD	
	24 h	48 h	24 h	48 h
**M**	3.0^a^ (3.0 ± 0.0)	3.0^a^ (3.0 ± 0.0)	2.0^a^ (1.80 ± 0.78)	2.5^a^ (2.50 ± 0.52)
**B**	4.0^a,b^ (4.0 ± 0.0)	4.0^a,b^ (3.90 ± 0.31)	4.0^b^ (3.9 ± 0.31)	4.0^b^ (4.0 ± 0.0)
**NP**	2.5^a,c^ (2.40 ± 0.69)	3.0^a,c^ (2.70 ± 0.48)	2.0^c^ (2.10 ± 0.31)	2.0^a^ (2.10 ± 0.56)
**ACQ**	2.0^a,c (+)^ (2.30 ± 0.67)	3.0^a,c (++)^ (2.70 ± 0.48)	4.0^b (+)^ (4.0 ± 0.0)	4.0 ^b (++)^ (3.90 ± 0.31)

Different letters ^(a,b,c)^ in columns mean significant differences between groups M, B, NP, and ACQ (*p* < 0.05; Kruskal-Wallis post hoc Dunn). Equal symbols in rows mean significant differences between cell types: ^(+)^ HGF-1 vs. SC-EHAD cultivated on ACQ disks at 24 h; ^(++)^ HGF-1 vs. SC-EHAD cultivated on ACQ disks at 48 h (*p* < 0.05; Kruskal-Wallis post hoc Dunn). No differences between 24 h and 48h were found in any of the groups (*p* > 0.05)

#### HGF-1

A higher prevalence of score 4 was observed at B compared to NP (median: 2.5) and ACQ (median: 2.0) surfaces at 24 h. No significant differences were found between groups B and M (median: 3.0). Similar results were observed after 48 hours and there were no significant intra-group differences between 24 and 48 hours (Table 3).

### Alizarin red staining

**Fig. 6:**
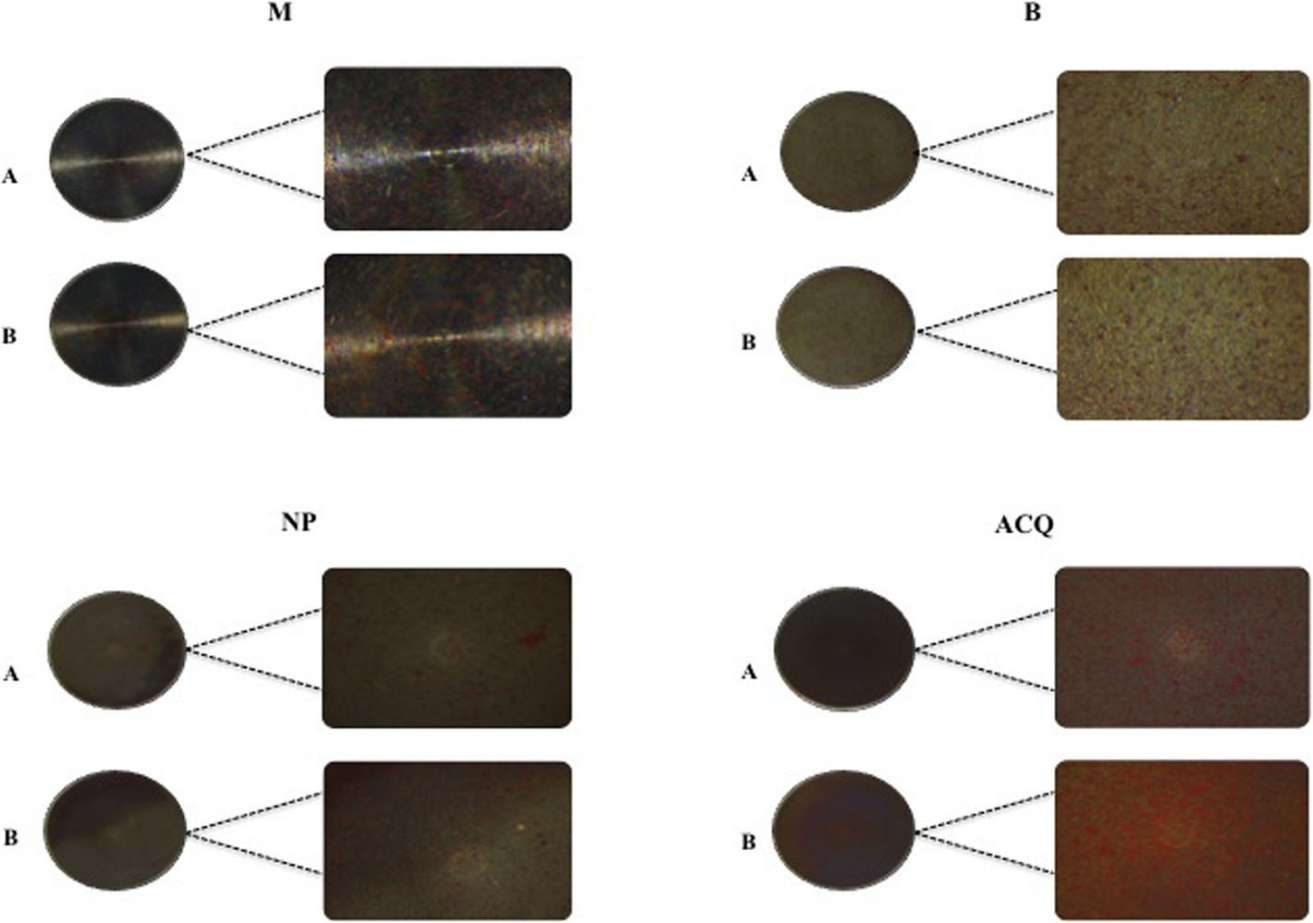
Mineralization activity of SC-EHAD cells at 14 days in the different groups. **a** Standard medium. **b** Osteogenic medium. *Squared images represent ¼ of disk surface

Mineralized nodules were observed at all surfaces at the different periods of investigation (Fig. 6). Comparative analysis between groups by Kruskal-Wallis post hoc Dunn showed significant differences in SC-EHAD cultivated on M (31.45% ± 1.51%) and ACQ (54.94% ± 4.80%) at 14 days. No significant differences were observed between groups at 28 days. Intra-group analysis by Wilcoxon test showed no differences in the percentage of mineralization observed at 14 and 28 days for any surface (Table 4).

**Table 4:** Percentage of mineralized area produced by SC-EHAD cells at the different surfaces, according to culture media and periods of investigation [median (mean ± standard-deviation)]

	14 days		28 days	
	DMEM	Osteogenic	DMEM	Osteogenic
M	31.56%^a^ (31.45% ± 1.51%)	35.64%^a^ (38.11% ± 4.29%)	37.20%^a^ (36.63% ± 1.12%)	36.05%^a^ (41.65% ± 10.23%)
B	48.69%^a,b^ (45.17% ± 6.61%)	45.69%^a^ (45.80% ± 4.45%)	52.18%^a^ (49.40% ± 6.54%)	54.92%^a^ (54.40% ± 3.84%)
NP	35.33%^a,b^ (34.38% ± 4.57%)	35.35%^a^ (36.97% ± 10.08%)	19.11%^a^ (26.68% ± 13.39%)	33.30%^a^ (41.59% ± 24.42%)
ACQ	56.16%^b^ (54.94% ± 4.80%)	62.93%^a^ (65.68% ± 17.52%)	57.71%^a^ (64.00% ± 20.69%)	51.55%^a^ (54.96% ± 10.49%)

Equal letters in columns represent no significant differences between groups; different letters in columns represent significant differences between groups (*p* < 0.05; Kruskal-Wallis post hoc Dunn)

### ALP activity

**Fig. 7:**
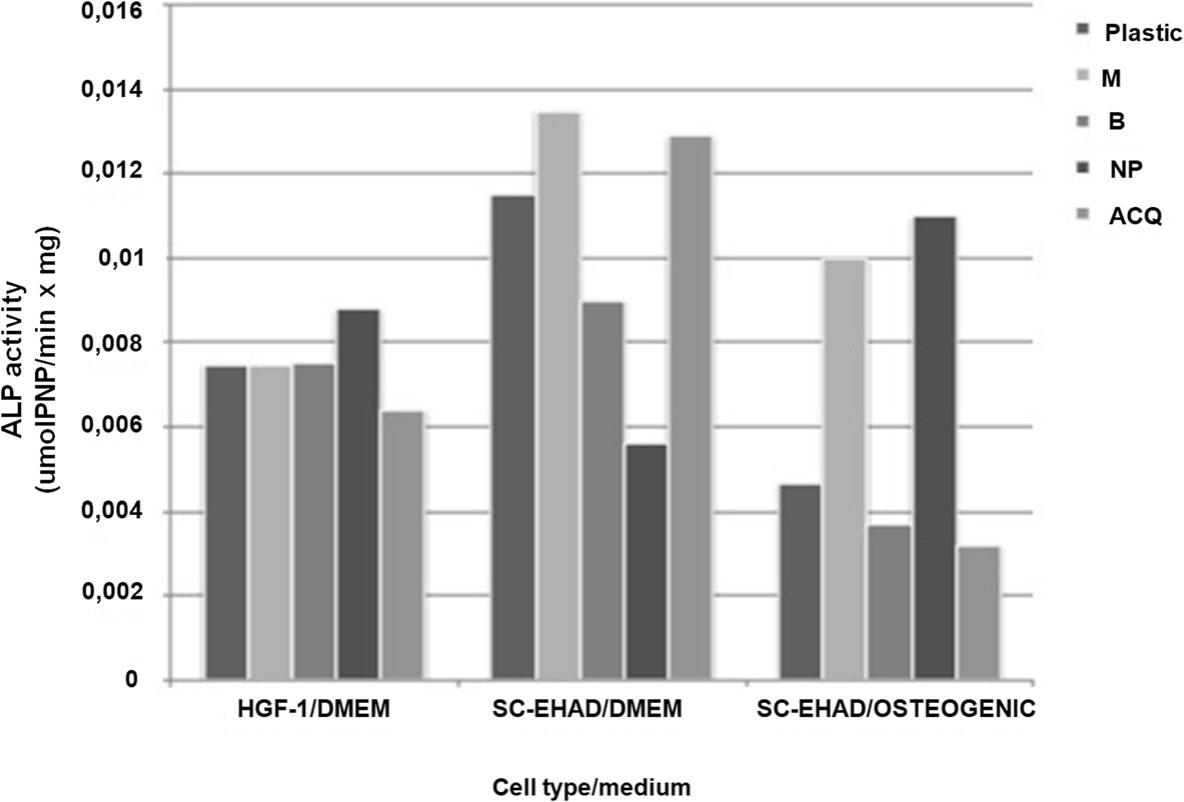
ALP activity of HGF-1 and SC-EHAD cells [DMEM (standard) and osteogenic media]

SC-EHAD and HGF-1 cells were expressed in all surfaces as well as on the plastic (negative control) (Fig. 7). SC-EHAD cells expressed increased ALP activity in osteogenic medium at M (213%) and NP (235.04%) surfaces, while decreased activity was observed at B (79.05%) and ACQ (68.37%) surfaces (Fig. 7). SC-EHAD cells cultivated on standard medium expressed a slightly increased ALP activity only at ACQ (112.17%), while M (90%), B (78.26%), and NP (48.86%) showed decreased ALP activity. HGF-1 cells expressed a slight decrease in ALP activity at ACQ (85.44%) surfaces and a slight increase at NP (117.49%), while similar ALP activity was observed at M (99.59%) and B surfaces (100.4%) (Fig. 7).

## Discussion

This study has investigated the response of osteoprogenitor and fibroblastic cells to different cp Ti surface treatments. According to the methods used, the adhesion and osteoblastic differentiation of SC-EHAD cells were influenced by moderately rough surfaces (B and ACQ). Hydrophilic surfaces (ACQ) were more likely to favor the adhesion of osteoblasts than fibroblasts. These findings are supported by increased osteogenic activity in vivo favoring earlier osseointegration [13, 30, 34].

Chemical composition of grade IV cp Ti disks was influenced by surface treatment. ACQ disks showed traces of Na and Cl at surface. Chemical treatment of titanium surfaces alters its surface properties, such as topography, structure, chemical composition, surface energy, and wettability, the last ones being related to a better surface-biological environment interaction [27].

Depending on surface roughness characteristics, cells may assume different phenotypes. Osteoblast-like cells express roughness-dependent phenotypic features, such as readily adhesion to rougher microtopography and a more differentiated morphology [12, 35]. A better adhesion of SC-EHAD cells were observed at ACQ hydrophilic surfaces, which could be associated to the stage of cell differentiation related to surface characteristics [33, 35–38]. Focal adhesion may be seen when a randomly rough surface is created by grit-blasting or acid subtraction; by the other side, some cells may see the grooves as a smooth surface, depending on the degree of roughness [38]. These might explain differences in studies, since roughness parameters vary from one study to another.

The stage of cell differentiation may be investigated by ALP and mineralization activity. The gene of ALP is an early marker of cell differentiation into osteoblasts that characterizes the beginning of mineralization activity, while bone sialoprotein gene is considered as a late marker [30, 37]. The expression of ALP increases with variations of surface microtopography, together with other proteins, such as bone sialoprotein, osteopontin, and osteocalcin, all of which are involved with the development of a bone [39].

The influence of NP and ACQ surfaces in the differentiation of immortalized osteoblasts was recently investigated [36] and suggested that ACQ resulted in decreased cell viability and adhesion compared with NP or plastic (negative control). On the other side, cells cultivated on ACQ surfaces expressed increased ALP activity and total protein, suggesting increased cell differentiation [36]. In agreement with our findings, also other studies [31, 33, 37, 40] using MSC cells have shown increased proliferation and higher ALP activity, bone sialoprotein expression, and mineralization, especially at an early observation period (14 days), on ACQ surfaces comparing with NP surfaces. Differences could be explained by the different cell lineages used in the studies. While Soares et al. [36] used an immortalized osteoblasts cell lineage, others [30, 40, 41] have used MSCs (mesenchymal stem cells). We have established a primary culture of bone granulation tissue removed from healing alveolar sockets 21 days after its creation, as previously described [27–29]. Studies have shown that the tissue present in healing sockets at the third to fourth week is rich in woven bone and immature osteoblasts [37, 42–45]. SC-EHAD cell characteristics suggest its osteoprogenitor nature [31].

Our findings suggest that ACQ and B surfaces exerted more influence in cell differentiation than the addition of β-glicerophosphate and ascorbic acid in conventional medium. Pivodova et al. [46] have also observed a decrease in the expression of ALP activity after 72 h, which could indicate that roughness parameters are important in cell behavior. Osteoblast-like cells decrease specific ALP activity as the surface roughness increases [35].

Different methods can be used to investigate surface roughness characteristics, including atomic force microscopy [47], confocal microscopy [47, 48], scanning electron microscopy [49, 50], optical profilometer [3, 49, 51], and light interferometer [50, 52]. Surface roughness is influenced by the topographic orientation (anisotropic or isotropic), with horizontal orientation determining a smoother surface although not influencing BIC or torque removal in rabbits’ tibiae [53]. Investigating surface characteristics at photomicrographs could eliminate variations related to the direction of surface roughness measurement. Although not ideal for tridimensional measurements, roughness parameters were investigated by the ImageJ plugin SurfCharJ, a software initially developed to the analysis of supercalendered papers in which surface representations are horizontally aligned by subtracting a regression plane from surface [54]. This software was found to be more suitable than other mathematical software for quantitative analysis of surface roughness of titanium alloys structures, providing information on global and local roughness analysis, gradient analysis, domain segmentation, surface leveling, and directional analysis [55].

To the current knowledge, dental implant surface characteristics were not investigated by this software. Due to technical problems during experimental procedures, roughness parameters were investigated by SurfCharJ. We found different values of roughness parameters from those reported in literature, which does not allow a direct comparison with other studies. However, it allows a comparison between groups to identify smoother (M) and rougher implants (NP). Additionally, no significant differences between groups were observed, except for Rsv parameter. These findings are in agreement with another study which, employing a different methodology, showed minimal differences in surface roughness between NP (Sa = 1.44 ± 1.15 μm) and ACQ (Sa = 1.26 ± 0.17 μm) surfaces [56]. No significant differences between groups can be found unless using high magnification (× 5000) images. At this magnification, a nanometric reticular surface was observed in hydrophilic surfaces [33]. According to manufacturer, ACQ surface is moderately rough, with Sa = 1.4–1.8 μm and Sz = 15 μm.

Our findings showed as well that HGF-1 with greater values of adhesion and proliferation on M and B surface. The surface geometry of smooth titanium discs could influence the behavior of fibroblast orientation and attachment of human gingival fibroblasts in vitro [57], and it is related with the mechanism of substratum curvature of fibroblasts cells orientations [58].

Within the limits of this study, it could be observed that moderately rough surfaces favor adhesion, proliferation, and differentiation of human osteoblastic progenitor cells as well as human gingival fibroblasts. Further, in vitro and in vivo studies are necessary to better evaluate surface treatment properties and its influence on osteogenesis and percentage of bone-to-implant contact.

## Conclusions

Moderately rough, hydrophilic surfaces of grade IV cp Ti disks influence osteoprogenitor cell adhesion, proliferation, and differentiation in osteoblasts.

## Data Availability

The authors from this work are available to support data.

## Abbreviations

cpTi: Commercially pure Titanium
M: Machined
B: Sandblasting
NP: Sandblasting and acid subtraction
ACQ: Hydrophilic treatment
SEM: Scanning electron microscopy
EDS: Energy dispersive x-ray spectrometry
SC-EHAD: Human surgically created early healing alveolar defects
HGF-1: Human gingival fibroblasts
ALP: Alkaline phosphatase
Rsk: Skewness of the assessed profile
Ra: Arithmetical mean deviation
Rq: Root mean square deviation
Rku: Kurtosis of the assessed profile
Rv: Lowest valley
Rp: Highest peak
Rt: Total height of the profile
DMEM: Eagle’s minimal essential medium
PBS: Phosphate buffered saline
FBS: Fetal bovine serum
HMDS: Hexametildisilazane
Ti: Titanium
Al: Aluminum
O: Oxygen
Na: Sodium
Cl: Chlorine
MSCs: Mesenchymal stem cells

## References

[1] Albrektsson T, Branemark PI, Hansson HA, Lindstrom J (1981). Osseointegrated titanium implants. Acta Orthop Scand.

[2] Albrektsson T, Wennerberg A (2005). The impact of oral implants – past and future, 1966 – 2042. J Can Dent Assoc..

[3] Liu R, Lei T, Dusevich V, Yao X, Liu Y, Walker MP, Wang Y, Ye L (2013). Surface characteristics and cell adhesion: a comparative study of four commercial dental implants. J Prosthod.

[4] Albrektsson T, Jacobsson M (1987). Bone–metal interface in osseointegration. J Prosthet Dent.

[5] Wennerberg A, Albrektsson T (2009). Effects of titanium surface topography on bone. Impl Res..

[6] Wennerberg A, Albrektsson T (2009). On implants surfaces: a review of current knwoledge and opinions. Int J Oral Maxillofac Implants..

[7] Mendonça G, Mendonça DBS, Simões LGP, Araújo AL, Leite ER, Duarte WR, Aragão FJL, Cooper LF (2009). The effects of implant surface nanoscale features on osteoblastspecific gene expression. Biomaterials.

[8] Anselme K (2000). The relative influence of the topography and chemistry of TiA16V4 surfaces on osteoblastic cell behaviour. Biomaterials.

[9] Anselme K (2000). Osteoblast adhesion on biomaterials. Biomaterials.

[10] Lange R, Luthen F, Beck U, Rychly J, Baumann A, Nebe B (2002). Cell–extracellular matrix interactions and physiochemical characteristics of titanium surfaces depend on the roughness of the material. Biomol Eng.

[11] Magnani A, Priamo A, Pasqui D, Barbucci R (2003). Cell behavior on chemically microstructured surfaces. Mater Sci Eng C.

[12] Boyan BD, Lossdorfer S, Wang L, Zhao G (2003). Osteoblasts generate an osteogenic microenvironment when grown on surfaces with rough microtopographies. Eur Cells Mat.

[13] Rupp F, Scheideler L, Rehbein D, Axmann D, Geis-Gerstorfer J (2004). Roughness induced dynamic changes of wettability of acid etched titanium implant modification. Biomaterials.

[14] Le Guehennec L, Lopez-Heredia MA, Enkel B, Weiss P, Amouriq Y, Layrolle P (2008). Osteoblastic cell behaviour on different titanium implant surfaces. Acta Biomater..

[15] Elias CN, Meirelles L (2010). Improving osseointegration of dental implants. Exp Rev Med Dev.

[16] Boyan BD, Schwartz Z, Lohmann CH, Sylvia VL, Cochran DL, Dean DD, Puzas JE (2003). Pretreatment of bone with osteoclasts affects phenotypic expression of osteoblast-like cells. J Orthop Res.

[17] Lüthen F, Lange R, Becker P (2005). The influence of surface roughness of titanium on b1 and b3-integrin adhesion and the organization of fibronectin in human osteoblastic cells. Biomaterials..

[18] Vallés G, Gil-Garay E, Munuera L (2008). Modulation of the cross-talk between macrophages and osteoblasts by titanium based particles. Biomaterials.

[19] Nebe B, Lüthen F, Lange R (2004). Topography-induced alterations in adhesion structures affect mineralization in human osteoblasts on titanium. Mater Sci Eng..

[20] Bagno A, Di Bello C (2004). Surface treatments and roughness properties of Ti based biomaterials. J Mater Sci Mater Med..

[21] Hazan R, Brener R, Oron U (1993). Bone growth to metal implants is regulated by their surface chemical properties. Biomaterials.

[22] Larsson C (1996). Bone response to surface-modified titanium implants: studies on the early tissue response to machined and electropolished implants with different oxide thicknesses. Biomaterials.

[23] Giavaresi G, Fini M, Cigada A, Chiesa R, Rondelli G, Rimondini L, Vicoli Aldini N, Martini L, Giardino R (2003). Histomorphometric and microhardness assessments of sheep cortical bone surrounding titanium implants with different surface treatments. J Biomed Mater Res.

[24] Kim MJ, Choi MU, Kim CW (2006). Activation of phospholipase D1 by surface roughness of titanium in MG63 osteoblast-like cell. Biomaterials.

[25] Albrektsson T, Sennerdy L, Wennerberg A (2008). State of the art of oral implants. Periodontology 2000.

[26] Albrektsson T, Buser D, Sennerdy L (2013). On crestal/marginal bone loss around dental implants. Int J Periodont Rest Dent..

[27] Schwarz F, Wieland M, Schwartz Z, Zhao G, Rupp F, Geis-Gerstorfer J, Schedle A, Broggini N, Bornstein M, Buser D, Ferguson SJ, Becker J, Boyan BD, Cochran DL (2009). Potential of chemically modified hydrophilic surface characteristics to support tissue integration of titanium dental implants. J Biomed Mater Res Part B: Appl Biomater.

[28] Bang S-M, Moon H-J, Kwon Y-D, Yoo J-Y, Pae A, Kwon IK (2014). Osteoblastic and osteoclastic differentiation on SLA and hydrophilic modified SLA titanium surfaces. Clin Oral Impl Res.

[29] Zhao GZO, Schwartz Z, Wieland M, Landolt D, Boyan BD (2006). Osteoblastlike cells are sensitive to submicron-scale surface structure. Clin Oral Implants Res.

[30] Mendonça G, Mendonça DBS, Aragão FJL, Cooper LF (2010). The combination of micron and nanotopography by H_2_SO_4_/H_2_O_2_ treatment and its effects on osteoblast-specific gene expression of hMSCs. J Biomed Mater Res.

[31] Sant’Ana ACP, Damante CA, Frias Martinez MA, Valdivia MAM, Karam PSBH, de Oliveira FA (2018). Isolation and characterization of progenitor cells from surgically created early healing alveolar defects in humans: a preliminary study. J Periodontol.

[32] Passanezi E, Janson WA, Nahás D, Campos A (1989). Newly forming bone autografts to treat periodontal infrabony pockets: clinical and histological events. Int J Periodontics Rest Dent..

[33] Sant'ana AC, Ferraz BF, de Rezende ML, Greghi SL, Damante CA, Passanezi E (2012). Newly forming bone graft: a novel surgical approach to the treatment of denuded roots. J Appl Oral Sci..

[34] Mendonça G, Mendonça DBS, Oliveira LS, Arújo CA. Effects of human mesenchymal stem cells on hydrophilic surfaces. ImplantNews 2013. 10(6 Part A):111–6 [In Portuguese].

[35] Martin JY, Schwartz Z, Hummert TW, Schraub DM, Simpson J, Lankford J, Dean DD, Cochran DL, Boyan BD (1995). Effect of titanium surface roughness on proliferation, differentiation, and protein synthesis of human osteoblast-like cells (MG63). J Biomed Mater Res.

[36] Soares PBF, Moura CCG, Coró CGC, Reis MVP, Zanetta-Barbosa D, Soares CJ (2013). Biological characterization of implant surfaces – in vitro study. Dental Mat.

[37] Bryington M, Mendonc AG, Nares S, Cooper LF (2014). Osteoblastic and cytokine gene expression of implant-adherent cells in humans. Clin Oral Impl Res..

[38] Lincks J, Boyan BD, Blanchard CR, Lohmann CH, Liu Y, Cochran DL, Dean DD, Schwartz Z (1998). Response of MG63 osteoblast-like cells to titanium and titanium alloy is dependente on surface roughness and composition. Biomaterials.

[39] Cooper LF, Zhou Y, Takebe J, Guo J, Abron A, Holmen A (2006). Fluoride modification effects on osteoblast behavior and bone formation at TiO2 grit-blasted c.p. titanium endosseous implants. Biomaterials.

[40] Olivares-Navarrete R, Hyzy SL, Hutton DL, Erdman CP, Wieland M, Boyan BD, Schwartz Z (2010). Direct and indirect effects of microstructured titanium substrates on the induction of mesenchymal stem cell differentiation towards the osteoblast lineage. Biomaterials.

[41] Bradford MM (1976). A rapid and sensitive method for the quantitation of microgram quantities of protein utilizing the principle of protein-dye binding. Anal Biochem.

[42] Evian CI, Rosenberg ES, Coslet JG, Corn H (1982). The osteogenic activity of bone removed from healing extraction sockets in humans. J Periodontol.

[43] Cardaropoli G, Araújo M, Hayacibara R, Sukekava F, Lindhe J (2005). Healing of extraction sockets and surgically produced – augmented and non-augmented – defects in alveolar ridge. An experimental study in dogs. J Clin Periodontol.

[44] Cardaropoli G, Araújo M, Lindhe J (2003). Dynamics of bone tissue formation in tooth extraction sites. J ClinPeriodontol.

[45] Penteado R, Romito GA, Pustiglioni FE, Marques MM (2005). Morphological and proliferative analysis of the healing tissue in human alveolar sockets covered or not by an e-PTFE membrane: a preliminary immunohistochemical and ultrastructural study. Braz J Oral Sci..

[46] Pivodova F, Frankova J, Dolezel P, Ulrichova J (2013). The response of osteoblast-like Saos-2 cells to modified titanium surfaces. Int J Oral Maxillofac Implants..

[47] Wennerberg A. On surface roughness and implant incorporation. Tese (Doutorado). Departmant of Biomaterial/Handicap Research, University of Götenborg. Götenborg, Sweden, 1996.

[48] Wennerberg A, Hallgren C, Johansson C, Danelli S (1998). A histomorphometric evaluation of screw-shaped implants each prepared with two surface roughnesses. Clin Oral Implants Res.

[49] Mustafa K, Wennerberg A, Wroblewski J, Hultenby K, Lopez BS, Arvidson K (2001). Determining optimal surface roughness of TiO(2) blasted titanium implant material for attachment, proliferation and differentiation of cells derived from human mandibular alveolar bone. Clin Oral Implants Res..

[50] Calvo-Guirado JL, Satorres-Nieto M, Aguilar-Salvatierra A, Delgado-Ruiz RA, de Val JEM-S, Gargallo-Albiol J, Gómez-Moreno G, Romanos GE (2015). Influence of surface treatment on osseointegration of dental implants: histological, histomorphometric and radiological analysis in vivo. Clin Oral Invest.

[51] Elias CN, Fernandes DJ, Resende CRS, Roestelb J (2015). Mechanical properties, surface morphology and stability of a modified commercially pure high strength titanium alloy for dental implants. Dental Mat.

[52] Gasik M, Braem A, Chaudhari A, Duyck J, Vleugels J (2015). Titanium implants with modified surfaces: meta-analysis of in vivo osteointegration. Mat Sci Engin C.

[53] Hallgren C, Sawase T, Ortengren U, Wennerberg A (2001). Histomorphometric and mechanical evaluation of the bone-tissue response to implants prepared with different orientation of surface topography. Clin Impl Dent Rel Res.

[54] Chinga G, Johnsen PO, Dougherty R, Berli EL, Walter J (2007). Quantification of the 3D microstructure of SC surfaces. J Microscopy.

[55] Safdar A. Microstructures and surface roughness of EBM produced Ti-6Al-4 V. Dissertation.Material Science, Malmö University, Malmö, Sweden, 2010.

[56] Sartoretto SC, Alves ATNN, Resende RFB, Calasans-Maia J, Granjeiro JM, Calasans-Maia MD (2015). Early osseointegration driven by the surface chemistry and wettability of dental implants. J Appl Oral Sci.

[57] Inoue T, Cox JE, Pilliar RM, Melcher AH (1987). Effect of the surface geometry of smooth and porous-coated titanium alloy on the orientation of fibroblasts in vitro. J Biomed Mater Res..

[58] Dunn GA, Heath JP (1976). A new hypothesis of contact guidance in tissue cells. Exp Cell Res.

